# Rethinking Digital Mental Health: From Nomophobia to Neurocognitive Exhaustion in LMIC Youth

**DOI:** 10.1002/hsr2.71278

**Published:** 2025-09-21

**Authors:** MD. Faisal Ahmed

**Affiliations:** ^1^ Department of Health Sciences and Informatics Bangladesh Institute of Innovative Health Research Mirpur Dhaka Bangladesh

To the Editor,

The recent article by Das et al. (2025) in *Health Science Reports* is a welcome contribution to the growing discourse on how digital lifestyles affect youth mental health, particularly in low‐ and middle‐income countries (LMICs) like Bangladesh [1]. Their findings—that young adult using social media for more than 4 h daily experience significantly higher levels of depression and anxiety—mark a critical inflection point in public health thinking. But we argue: this is just the tip of the iceberg.

Our own study, recently published in the *Journal of Public Health*, shifts the conversation further—into the behavioral dependency syndrome of nomophobia (the fear of being without a mobile phone). Among 701 Bangladeshi university students, nearly half (47.8%) experienced severe nomophobia, which was significantly associated with reduced psychological well‐being and greater insomnia severity [2]. While time spent online is an issue, the psychological and physiological *entanglement* with our devices may be far more dangerous—and insidious.

Digital dependency is no longer simply about “screen time.” It is about neurocognitive fatigue, emotional deregulation, and identity tethering to mobile technology—what we call the Digital Dependency–Distress Cascade (Figure 1).

**Figure 1:**
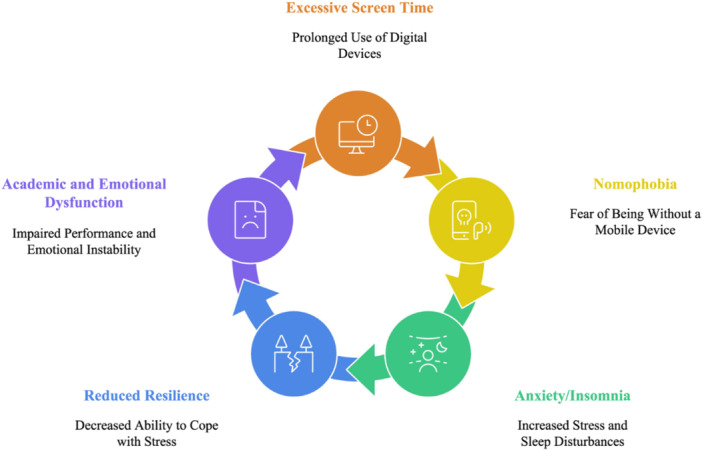
Digital dependency–distress cascade: A proposed model of psychosocial and cognitive consequences of excessive screen engagement in youth (*Source:* Author's own creation).

This cascade is well‐documented beyond Bangladesh. In Thailand, Kaewpradit et al. (2025) found that 48.4% of university students reported Excessive Digital Screen Time (EDST), primarily via smartphones and tablets, strongly associated with mental distress and poor time management—especially among health science students [3]. In Spain, Bernabé‐Mateo et al. (2025) reported that female nursing students with poor diets, poor sleep, or food addiction were at significantly higher risk of nomophobia, directly impairing both health and professional readiness [4]. In our own region, Ahmed (2024) found that 92.3% of Bangladeshi university students suffer from poor sleep, with moderate internet addiction most common—indicating a silent, systemic collapse of sleep hygiene and emotional regulation [5].

Together, these studies reveal a shared global crisis: digital dependency is rewiring youth cognition and destabilizing emotional homeostasis. In LMICs, where healthcare access is limited and psychological literacy is low, this crisis becomes existential. The traditional public health narrative has treated digital behavior as a lifestyle variable. We argue this is no longer sufficient. We need to treat nomophobia, screen addiction, and insomnia as interconnected syndromes of a neurobehavioral epidemic. These issues are not mild distractions—they are developmental disruptors with the power to alter sleep cycles, academic functioning, relationship dynamics, and long‐term productivity. There is also a gendered and socioeconomic gradient. Young women in Spain and Bangladesh suffer higher digital anxiety [2, 4]. In Bangladesh, youth from higher SES backgrounds have higher nomophobia, while those from lower SES backgrounds suffer poorer sleep quality—splitting the burden across lines of privilege and precarity [2, 5].

We urge researchers and policymakers in LMICs to:

- Recognize nomophobia and EDST as clinical risk factors—not just behavioral patterns.
- Introduce universal digital literacy programs, especially in universities.
- Screen for nomophobia and sleep disorders in student health checkups.
- Implement institutional digital detox periods, linked with positive psychology, mindfulness, and resilience training.
- Reform academic structures to reward healthy digital use, not constant availability.

This is not a call for digital abstinence—but for cognitive sovereignty. Youth must reclaim attention, agency, and emotional boundaries in a hyperconnected world. Bangladeshi youth—and their global peers—are not merely “online too much.” They are living inside their devices, often to the detriment of mental health, sleep, and identity stability. Studies like Das et al. provide the first warning signs [1]. Our work, and that of colleagues worldwide, shows that we are deep into the second wave: psychological dependence and neurocognitive fallout [2, 3, 4, 5].

Let us act—not tomorrow, but now. Because the question is no longer: *How much time are youth spending online?*

The question is: *How much of themselves are they leaving behind?*

## Author Contributions

**MD Faisal Ahmed:** conceptualization, investigation, funding acquisition, writing – original draft, writing – review and editing, visualization, project administration, data curation, software, methodology, validation, formal analysis.

## Conflicts of Interest

The author declares no conflicts of interest.

## Transparency Statement

The Corresponding author (MFA) affirms that this manuscript is an honest, accurate, and transparent account of the study being reported; that no important aspects of the study have been omitted; and that any discrepancies from the study as planned (and, if relevant, registered) have been explained.

## Data Availability

No new data were generated or analyzed in this study. All supporting materials and references are publicly available as cited in the article.
